# Pharmacists’ Attitudes and Perceived Barriers to Human Papillomavirus (HPV) Vaccination Services

**DOI:** 10.3390/pharmacy5030045

**Published:** 2017-08-07

**Authors:** Tessa J. Hastings, Lindsey A. Hohmann, Stuart J. McFarland, Benjamin S. Teeter, Salisa C. Westrick

**Affiliations:** 1 Health Outcomes Research and Policy, Harrison School of Pharmacy Auburn University, 020 James E. Foy Hall, Auburn, AL 36849, USA; tjh0043@auburn.edu (T.J.H.); lah0036@auburn.edu (L.A.H.); 2 College of Medicine, University of South Alabama, 307 N University Blvd, Mobile, AL 36688, USA; sjm1721@jagmail.southalabama.edu; 3 Department of Pharmacy Practice, College of Pharmacy, University of Arkansas for Medical Sciences, 4301 W Markham St, Little Rock, AR 72205, USA; BSTeeter@uams.edu

**Keywords:** human papillomavirus, community pharmacy, cervical cancer, adolescent vaccination

## Abstract

Use of non-traditional settings such as community pharmacies has been suggested to increase human papillomavirus (HPV) vaccination uptake and completion rates. The objectives of this study were to explore HPV vaccination services and strategies employed by pharmacies to increase HPV vaccine uptake, pharmacists’ attitudes towards the HPV vaccine, and pharmacists’ perceived barriers to providing HPV vaccination services in community pharmacies. A pre-piloted mail survey was sent to 350 randomly selected community pharmacies in Alabama in 2014. Measures included types of vaccines administered and marketing/recommendation strategies, pharmacists’ attitudes towards the HPV vaccine, and perceived system and parental barriers. Data analysis largely took the form of descriptive statistics. 154 pharmacists completed the survey (response rate = 44%). The majority believed vaccination is the best protection against cervical cancer (85.3%), HPV is a serious threat to health for girls (78.8%) and boys (55.6%), and children should not wait until they are sexually active to be vaccinated (80.1%). Perceived system barriers included insufficient patient demand (56.5%), insurance plans not covering vaccination cost (54.8%), and vaccine expiration before use (54.1%). Respondents also perceived parents to have inadequate education and understanding about HPV infection (86.6%) and vaccine safety (78.7%). Pharmacists have positive perceptions regarding the HPV vaccine. Barriers related to system factors and perceived parental concerns must be overcome to increase pharmacist involvement in HPV vaccinations.

## 1. Introduction

Human papillomavirus (HPV) infection is an important public health issue. Almost 80 million people are infected with HPV in the United States, and most sexually active individuals will contract some form of HPV during their lifetime [1]. HPV strains 16 and 18 cause over 70% of cervical cancer cases [2]. Three types of HPV vaccine are currently approved in the U.S. to prevent infection with these HPV strains: Gardasil^®^ (for girls and boys age 9–26 years), Gardasil 9^®^ (for girls age 9–26 years and boys age 9–15 years), and Cervarix^®^ (for girls age 9–25 years) [2]. In addition to strains 16 and 18, Gardasil^®^ and Gardasil 9^®^ protect against several additional less common cancer-causing strains [2]. Guidelines recommend administration of one of these vaccines as a three-dose series beginning at 11 years of age [2]. Despite this recommendation, adolescent HPV vaccination rates remain low. Globally, it is estimated that 59 million women (50.1%) receive at least one dose of HPV and 47 million (39.7%) complete the three-dose series [3]. In the United States in 2015, 4 out of 10 adolescent girls and 5 out of 10 adolescent boys had never received a single dose of the HPV vaccine [4]. Furthermore, completion of the three-dose series falls far below the 80% United States national objective, with only 41.9% of girls and 28.1% of boys vaccinated with three doses in 2015 [4]. This low vaccination rate is concerning for the prevention of cervical cancer in the United States, especially in southern states, where vaccination rates are disproportionately low but where vaccines are needed most, suggested by a higher than average incidence of cervical cancer [5,6]. In Alabama, 40.8% of girls and only 22.6% of boys completed the three dose series in 2015 [4].

Barriers to initiation and completion of the HPV vaccine series reported in existing literature include lack of recommendation by primary care providers, cost, insurance coverage, necessity of multiple visits to primary care providers, and parental concerns [7]. Also, about one-third of adolescents age 13–17 years old had no preventive care visits with their physicians, creating a lack of opportunity to recommend the HPV vaccine [8]. Innovative methods to overcome these barriers and to promote the HPV vaccine among hard-to-reach adolescents are needed to improve HPV vaccination and completion rates. One method that has been suggested is the use of non-traditional settings such as community pharmacies, as they are easily accessible with longer hours of operation and no need for appointments, unlike traditional settings such as a physician’s office [9]. In addition to the convenience and accessibility offered by pharmacies, the literature shows that parents and adolescents support pharmacy-based provision of vaccinations [9,10,11].

Pharmacists are increasingly becoming accepted as immunization providers for adult vaccinations by patients, physicians, and national organizations in the United States [12], and globally [13,14,15,16]. Currently in the United States, all 50 states permit pharmacists to administer vaccines [17]. Many states include the HPV vaccine within this authority; however, the specific requirements and limitations vary widely by state [17]. Pharmacists in Alabama are permitted to administer any vaccine to patients with no age restrictions [18]. However, for some cases, such as the administration of the HPV vaccine, pharmacists are required to obtain a prescription from a licensed prescriber [18]. Despite wide acceptance among adults, little is known about adolescent vaccination services in US community pharmacies, especially for the HPV vaccine. The objectives of this study were to explore: (1) the extent to which HPV vaccination services are currently being offered in community pharmacies as well as strategies to increase the uptake of the HPV vaccine; (2) pharmacists’ attitudes towards the HPV vaccine; and (3) perceived barriers to the provision of HPV vaccination services in community pharmacies.

## 2. Materials and Methods

### 2.1. Study Design and Sample

This study utilized a cross-sectional survey of community pharmacies in Alabama. The unit of analysis was at the pharmacy level. One key informant represented each pharmacy; they included pharmacy owners, managers, or staff pharmacists. All procedures were approved by the first author’s Institutional Review Board as an expedited review. 

The sampling frame used in this study to select community pharmacies was Hayes’ Directory, a database of community pharmacies in the United States, which provided name, mailing address, county name, telephone number, and fax number for each community pharmacy. Pharmacies that did not serve the typical public (i.e., walk-in customers) or dispense medications were excluded from participation. Pharmacies were not required to provide HPV vaccination services in order to participate in this study. Of the 1176 community pharmacies in Alabama, 350 community pharmacies were randomly sampled. The decision to invite 350 pharmacies was made after careful consideration of the balance between survey costs and survey errors. Based on our previous survey studies with community pharmacies, we anticipated response rates to fall between 30% and 40%, which would result in an expected sample size of 105–140 [19,20]. Setting a confidence level of 95%, a sample size of 350 would yield a margin of error range between 7.4% and 8.8%, which was deemed acceptable.

The survey was administered from June to August 2014 based on a modified version of Dillman’s Tailored Design Method [21]. To maximize the response rate and minimize the likelihood of non-response bias, four methods of contact were employed including a pre-notification postcard, a survey packet, a reminder postcard, and a replacement survey packet; all were delivered via first-class mail and addressed to the pharmacist. A web address was provided on all contacts that led to an online version of the survey for those who preferred to complete the survey electronically. To ensure that multiple pharmacists from one location did not complete the survey, a unique identifier was assigned to each pharmacy, which was required to access the electronic survey. Survey packets and replacement packets contained an invitation letter, information letter, survey, and pre-addressed, stamped return envelope. The invitation letter included in this packet briefly explained the purpose of this study, how to participate, and the completion deadline. Details regarding study participation were outlined in the information letter including risks, benefits, compensation, and confidentiality. Each respondent received a $20 incentive after receipt of their completed survey. To maintain confidentiality, the last page of the survey containing contact information for payment purposes was separated from the survey packet upon receipt. Respondents were made aware that findings would be reported in aggregated form and that identifiable data would be kept confidential and only accessed by the research team.

### 2.2. Survey Variables and Measures 

The survey was comprised of 65 questions and required approximately 15 min to complete. Measures can be categorized into five sections: (1) key informant and pharmacy site demographic characteristics; (2) general vaccination services and strategies used to increase HPV vaccine uptake; (3) pharmacists’ perceptions of HPV and the vaccine; (4) perceived system barriers to the provision of HPV vaccinations; and (5) perceived parental reasons for HPV vaccine hesitancy. The majority of questions were formatted as 5-point Likert-type rating scales. For example, *“How much do you agree or disagree with the following statements about the parents of teens and adolescents and their perceptions regarding HPV vaccine?”* with answer choices ranging from strongly disagree to strongly agree. Survey questions assessing vaccination services and strategies to increase uptake were informed by our prior research in vaccination services, while items measuring perceptions of HPV and the vaccine were modified from an existing instrument utilized by Khan and colleagues [22]. To assess system barriers, previous research informed the survey items [23,24,25]. Lastly, an existing measure developed by Luque and colleagues to assess perceived parental reasons for vaccine hesitancy was used [23]. 

### 2.3. Pre-Testing

The survey questions were written and refined by the research team. To ensure the content validity of survey questions, the research team consulted with a licensed pharmacist. After changes were made, the survey was pre-tested with 6 community pharmacists in Alabama in order to assess the face validity of the included measures. Based on their feedback, minor modifications to the formatting and answer choices were made to improve clarity. Due to their participation in pre-testing of the survey questions, these 6 pharmacists were excluded from the sampling frame prior to selecting the random sample. 

### 2.4. Non-Response Bias Investigation 

A non-response investigation was conducted after survey completion to determine if respondents’ demographic characteristics and the vaccination services they offered differed significantly when compared to non-respondents. Community pharmacies that did not respond to survey requests were randomly selected and contacted via telephone. Using a five-minute structured telephone interview, scripted interview questions gathered information regarding key informant and pharmacy site demographics as well as general vaccination services provided. Non-respondents’ data was aggregated and compared to that of respondents to assess any differences. 

### 2.5. Data Analysis 

All data analysis was performed using IBM SPSS Statistics version 22 software (IBM Corp., Armonk, NY, USA). Descriptive statistics were used to describe respondent characteristics, current vaccination practices, attitudes, and barriers. Comparison of non-respondents and respondents to investigate non-response bias was completed using one-way analysis of variance for continuous variables and chi-square for categorical variables. A significance level of 0.05 was used for all statistical analyses.

## 3. Results

### 3.1. Demographics and Non-Response Bias Investigation 

A total of 154 pharmacies completed the survey (44% response rate). Table 1 displays the demographics of respondents and their pharmacies. The majority of key informants were female (57.5%), held a PharmD degree (50.6%), were employed as pharmacy managers (54.5%), and were trained in vaccine administration (80.8%). Thirty pharmacies were randomly sampled in the non-response bias investigation, of which 18 responded. There were no significant differences found between respondents and non-respondents in terms of individual and site demographics. Additionally, there were no significant differences found in the number of vaccine types offered in the past 12 months between respondents and non-respondents. 

### 3.2. HPV Vaccination Services and Strategies Employed to Increase HPV Vaccine Uptake 

Table 2 details the number of pharmacies providing vaccinations and the strategies employed to increase vaccine uptake. A total of 113 responding pharmacies (73.4%) reported offering at least one vaccination in the previous 12 months. Of these pharmacies, influenza was the most common vaccine provided (94.7%), followed by herpes zoster (83.2%), pneumococcal polysaccharide (PPSV23) (53.1%), and tetanus/diphtheria/pertussis (Tdap) (42.5%). Among those that provided at least one type of vaccine, most (82.6%) did not encounter any patients requesting information about the HPV vaccine. Further, 89% of pharmacies had not made any recommendations to male or female patients or parents of patients regarding the need for the HPV vaccine. Over 90% of pharmacies also indicated that they did not refer patients to places other than the pharmacy to obtain the HPV vaccine. Among the 9 pharmacies that did report referring patients to another location for the HPV vaccine, the most commonly mentioned site was a pediatrician’s office. 

Of the 18 pharmacies that reported having the HPV vaccine available in stock, only 5 pharmacies had actually administered the HPV vaccine, averaging less than 2 doses in the past year. Three of the five pharmacies that provided the vaccine in the past 12 months did so through a written protocol with a physician, while another stated that they contacted a physician they had worked with in the past in order to obtain a prescription for the patient allowing them to administer the HPV vaccine. The fifth pharmacy administered the HPV vaccine only after the patient obtained and delivered a written prescription from his or her physician. Although only 18 pharmacies reported having HPV vaccine in stock and only 5 had actually administered the vaccine in the past 12 months, 36 (31.9%) indicated that they planned to offer or continue offering the vaccine in the next 12 months.

It is worth noting the various marketing strategies the 5 pharmacies that administered the HPV vaccine in the past 12 months employed. Methods reported to market HPV vaccinations included: general flyers accompanying dispensed prescriptions, billboards, posters at the pharmacy, and generic telephone messages. When asked if they used a system to identify patients who were eligible for their first dose of HPV vaccine, 3 of 5 said no; they did not use any personalized methods to market their HPV vaccination services. When comparing general vaccination strategies used by those pharmacies that had administered the HPV vaccine in the past 12 months to those that had not, the proportion using flyers, generic telephone messages, billboards, and posters were similar (Table 3). Pharmacies that had not administered the HPV vaccine in the past 12 months reported employing additional strategies including newspapers (33.3%) and radio announcements (26.7%). 

### 3.3. Pharmacists’ Attitudes towards HPV and the Vaccine

Table 4 shows pharmacists’ attitudes towards HPV and the HPV vaccine. A large percentage (47.3%) of pharmacists strongly agreed that vaccination against HPV is the best protection against cervical cancer. Over 78% percent agreed that HPV infection is a serious threat to a girl’s health, but a much lower proportion (55.6%) felt the same about HPV’s threat to a boy’s health. Over half of respondents (52.7%) agreed that the optimal age to have a child vaccinated against HPV is 11–12 years. Further, 43.7% agreed that vaccinated children will not practice riskier sexual behaviors. No differences were found in pharmacists’ attitudes toward HPV/HPV vaccine based on demographic factors (gender, number of years practicing as a pharmacist, or vaccine administration training).

### 3.4. Barriers to Providing HPV Vaccinations 

Pharmacists reported a number of perceived system barriers to providing HPV vaccination services (Table 5). Over half (56.5%) of respondents reported that a lack of patients who want the HPV vaccine was extremely or very likely to be a factor preventing their pharmacy from providing HPV vaccination services. Other factors perceived to be very/extremely likely to be barriers include: the failure of some insurance companies to cover the cost of the vaccination (54.8%); the vaccine expiring before use (54.1%); difficulty ensuring that patients complete the necessary three doses (39.9%); and lack of adequate reimbursement (38.4%).

Table 6 reports respondents’ perceived parental reasons for HPV vaccine hesitancy. The majority of respondents believed that parents lack adequate education about the HPV infection (86.6% somewhat or strongly agree). Parental concerns about the safety (78.7%), reluctance to discuss sexuality/sexually transmitted infections (76%), concerns that the vaccine condones premarital sex (67.3%), beliefs that their child is not at risk (67.3%), beliefs that their child is too young (65.3%), concerns about efficacy (64.6%), concerns about children practicing riskier sex behaviors (58.7%), and cost (53.3%) were also found to be perceived barriers. No differences were found in pharmacists’ perceived barriers to HPV vaccination based on demographic factors (gender, number of years practicing as a pharmacist, or vaccine administration training). 

## 4. Discussion

HPV vaccination rates are disproportionately low in the Southern United States, which is especially concerning due to the higher rate of cervical cancer in this region when compared to other regions of the U.S. [5]. Utilizing community pharmacies for the provision of the HPV vaccine may be a method to increase vaccination rates, especially in rural areas where access to vaccine providers is limited. However, this study shows that the HPV vaccine provision in community pharmacies is low. Of pharmacies providing vaccinations, we found that only 11.7% had the HPV vaccine in their inventory. This rate in Alabama is much lower than the national survey’s results which reported that 37% of U.S. pharmacies provide the HPV vaccine [26]. This lower rate could be attributed to several system barriers such as perceived lack of patient interest, insufficient reimbursement, and perceived parental hesitancy. Results from the study show that marketing strategies did not differ markedly between pharmacies that administer the HPV vaccine and those that do not. As 68.1% of pharmacists reported that they do not plan to offer or continue offering the vaccine in the next year, future research should demonstrate successful HPV vaccine services in community pharmacies and outline strategies to overcome system barriers and parental hesitancy. 

Pharmacists generally reported positive perceptions of the HPV vaccine; however, there were many perceived system barriers identified that could be limiting the provision of this vaccine in the community pharmacy setting. Some of these barriers are related to the vaccine itself while others are related to coverage and reimbursement for the vaccine. Lack of reimbursement has been identified in previous research as a barrier to general vaccine provision [27,28]. Countries such as the United Kingdom and Australia, which are able to overcome this reimbursement barrier due to differences in the structure of their health care system, have achieved much higher HPV vaccination uptake when compared to the United States. The United Kingdom and Australia reported HPV vaccination rates among girls to be 60.4% and 71.2% respectively [29]. Previous research has also identified the need to acquire a prescription from a physician to administer the vaccine as a challenge [17]. This did not appear to be a factor affecting the decision to offer the vaccine among the majority of pharmacists in this study, perhaps because other barriers overwhelm them.

Overall, the results of this study show that a greater proportion of pharmacists rated perceptions of parental concerns and hesitancy as likely barriers to a greater extent when compared to the system-related factors. This suggests that while system factors may hinder the provision of HPV vaccination services, pharmacists’ perceptions of parental concerns and hesitancy may be more of a factor in impeding pharmacists’ from making recommendations to parents. This is not surprising, as many system-related barriers may have already been addressed in the development of immunization services for other vaccinations. Looking specifically at parental concerns, the majority of pharmacists believe that parents lack adequate understanding regarding the HPV infection in general. This perception may be valid, as studies in southern regions have reported that parents have limited knowledge and awareness of the HPV infection and HPV vaccine [30,31]. Overall, the majority of pharmacists believed that parents’ perceptions of the HPV infection and vaccine were barriers to teens and adolescents receiving this vaccine. Additionally, the majority of responders believe that the optimal age to be vaccinated against HPV is 11–12. Because of the young age of vaccine recipients, pharmacists may perceive that parents would not be ready for pharmacists to provide the HPV vaccine to their adolescent children; this belief is consistent with the findings reported by Westrick and colleagues [9]. Hence, addressing system factors may be helpful but will likely be unsuccessful unless pharmacists’ perceived parental barriers are overcome. One method to overcome these barriers may include training pharmacy staff to be confident and proactive in educating and recommending the HPV vaccine to parents. Receiving positive responses from parents could help improve their perceptions of parental concerns and motivate them to recommend the vaccine to the parents of adolescents. 

Research has shown that provider recommendations are a key factor influencing vaccine uptake [7]. However, we found that pharmacists in this study did not recommend the HPV vaccine. Such missed opportunities have been successfully reduced via the use of provider reminder systems [32]. Thus, to bring about change in community pharmacies, pharmacists’ recommendations may be facilitated by installation of reminder systems in pharmacy dispensing software to aid in identifying vaccine-eligible patients. Potential reminder strategies may include posters in the pharmacy, immunization registry-based reminders and age-based “flags” in patients’ prescription bags to alert pharmacists to vaccination opportunities, or ready-made forms to quickly allow pharmacists or technicians to make personalized recommendations while counseling patients or at prescription drop-off, respectively [32,33,34,35]. Two of the five pharmacies administering the HPV vaccine in this study had implemented systems such as these to identify eligible patients. These strategies require minimum time-commitment from the pharmacist and pharmacy staff, and have been shown to increase patients’ commitment to receive vaccination services at a community pharmacy [33]. Buy-in and referrals from local physicians are also important and may be achieved through letters to physicians’ offices highlighting pharmacy vaccination services [32]. Buy-in could also be established through collaborative practice agreements; three of the five pharmacies administering the HPV vaccine in this study did have a written protocol. These physicians may partner with pharmacies such that the first dose of the HPV vaccine is administered at the physician’s office and the physician writes a prescription for the second and third doses to be obtained at a community pharmacy [32]. This strategy may ultimately save physicians’ and nurses’ time, maximize pharmacies’ profitability, and increase convenience for the patient due to pharmacies’ extended hours of operation. 

This study has several limitations that must be considered. First, although the overall response rate was 44% (154 pharmacies out of 350 sampled), only 5 respondents (3.2%) reported administering the HPV vaccine in the past 12 months, which might limit meaningful interpretation of pharmacies’ marketing strategies to increase HPV vaccinations. HPV vaccine specific marketing strategies used by respondents who had not administered the HPV vaccine in the past 12 months were not assessed. It is possible that these pharmacies were using HPV vaccine specific marketing strategies but that they were not successful. Future research should further examine marketing strategies in greater detail, including variations in success for specific vaccines. Furthermore, identification of potential vaccine recipients is the first step. As such, future research should assess how pharmacists identify potential recipients and their confidence in doing so. While this study assessed pharmacists’ attitudes toward the HPV vaccine, pharmacists’ confidence in speaking to parents and patients about the vaccine was not addressed. This is an area of future study that will be necessary for the development of suggested educational training programs. Additionally, a greater proportion of key informants were female (57.5%) and employed as pharmacy managers (54.5%). Thus, the study’s findings may not be generalizable to all pharmacists in Alabama. Also, the study’s population of interest was community pharmacies in Alabama, and therefore results may not be generalizable to community pharmacies in other countries and areas of the United States. As this study collected self-reported attitudes and behaviors, social-desirability and recall bias may be a concern. Finally, individuals who responded to the survey may differ from non-respondents. However, no statistically significant differences were found in the non-response bias investigation. 

## 5. Conclusions

Pharmacists have positive attitudes regarding provision of the HPV vaccine to teens and adolescents. However, system factors and perceived parental concerns may prevent pharmacists from engaging in the provision of the HPV vaccination in community pharmacies. Overcoming these barriers is necessary to increase pharmacist involvement in HPV vaccinations. Strategies to do so may include pharmacy staff training, reminder systems, and physician partnerships among others.

## Figures and Tables

**Table 1:** Respondent and Pharmacy Characteristics.

Characteristics	Number (%)
**Sex** (*N* = 153)	
Male	65 (42.5)
Female	88 (57.5)
**Education** (*N* = 154)	
PharmD	78 (50.6)
B.S. Pharmacy	73 (47.4)
Residency	5 (3.2)
Masters	1 (0.6)
Other	3 (1.9)
**Title** (*N* = 154)	
Pharmacy Manager	84 (54.5)
Staff Pharmacist	45 (29.2)
Owner/Partner	33 (21.4)
Other	2 (1.3)
**Trained in Vaccine Administration** (*N* = 151)	
Yes	122 (80.8)
No	29 (19.2)
**Type of Pharmacy** (*N* = 153)	
Chain Pharmacy	81 (52.9)
Independently Owned Pharmacy	72 (47.1)
**Hours Open per Week** (*N* = 154)	
Less than 40 h	3 (1.9)
40–49 h	17 (11.0)
50–59 h	47 (30.5)
60–69 h	18 (11.7)
70–79 h	48 (31.2)
80 or more hours	21 (13.6)
**Average Prescription Volume per Day** (*N* = 153)	
Less than 100	15 (9.8)
100–199	58 (37.9)
200–299	39 (25.5)
300–399	19 (12.4)
400+	22 (14.4)
	**Mean (SD)**
**Number of Years Practicing as a Pharmacist** (*N* = 153)	16.8 (13.5)
**Number of Years at Current Pharmacy** (*N* = 151)	7.5 (7.6)
**Number of Staff Pharmacists Employed (FTE)** (*N* = 152)	2.1 (1.1)
**Number of PharmD Staff Pharmacists Employed (FTE)** (*N* = 146)	1.1 (.9)
**Number of Technicians Employed (FTE)** (*N* = 153)	3.8 (2.2)
**Number of Pharmacists Trained in Vaccine Administration** (*N* = 153)	1.8 (1.1)
**Number of Pharmacists Actively Administering Vaccines** (*N* = 153)	1.7 (1.2)
**Number of Vaccine Types Available in the Past 12 Months** (*N* = 113)	3.55 (3.5)

**Table 2:** Vaccination Services and Strategies Utilized to Increase Human Papillomavirus Vaccinations.

Characteristics	No. (%)
**Vaccination services offered in the past 12 months**	
Influenza	107 (94.7)
Herpes zoster	94 (83.2)
Pneumococcal polysaccharide (PPSV23)	60 (53.1)
Tetanus/Diphtheria/Pertussis (Tdap)	48 (42.5)
Pneumococcal 13-valent conjugate (PCV13)	39 (34.5)
Hepatitis B	35 (31.1)
Meningococcal	33 (29.2)
Travel vaccines (yellow fever, typhoid, etc)	28 (24.8)
Hepatitis A	27 (23.9)
Measles, mumps, rubella (MMR)	23 (20.4)
Tetanus/Diphtheria (Td)	23 (20.4)
Human papillomavirus (HPV)	20 (17.7)
Varicella	11 (9.7)
Other	2 (1.8)
**Patients requested information about the HPV vaccine in the past 12 months** (*N* = 109)	
Yes	19 (17.4)
No	90 (82.6)
**Recommended HPV vaccine to male and female patients or parents of male and female patients in the past 12 months (*N* = 110) ^a^**	

Male patients 11–12 years	3 (2.7)
Male patients 13–18 years	3 (2.7)
Male patients 19–26 years	2 (1.8)
Female patients 11–12 years	5 (4.5)
Female patients 13–18 years	3 (2.7)
Female patients 19–26 years	5 (4.5)
No recommendations have been made	98 (89)
**Referred patients to other places for HPV vaccine in the past 12 months (*N* = 113)**	
Yes	9 (8.0)
County Health Department	1 (11.1)
Physician in general	2 (22.2)
OBGYN specifically	1 (11.1)
PCP or gynecologist	1 (11.1)
Pediatrician	4 (44.4)
No	104 (92)
**HPV vaccine administered in the past 12 months (*N* = 113)**	
Yes	5 (4.4)
Per written protocol with physician	3 (60)
Patients obtain and bring in written prescription from physician	1 (20)
Pharmacy contacts other known physician/physician co-worker to obtain prescription	1 (20)
No	108 (95.6)
**Plans to offer/continue offering HPV vaccine in the next 12 months (*N* = 113)**	
Yes	36 (31.9)
No	77 (68.1)
	**Mean (SD)**
**Number of HPV vaccine doses administered in the past 12 months (*N* = 5)**	1.4 (0.55)

^a^ Participants were instructed to choose all applicable categories.

**Table 3:** Comparison of General Marketing Strategies Used by Pharmacies who had and had not Administered the HPV Vaccine in the Past 12 Months ^a^.

	Pharmacies with HPV Vaccine in Stock (*N* = 20)	
General Marketing Strategies	Administered HPV Vaccine in the Past 12 Months (*N* = 5)	Did not Administer HPV Vaccine in the Past 12 Months (*N* = 15)
Newspapers	0	5 (33.3%)
Radio announcements	0	4 (26.7%)
Flyers accompanying prescriptions dispensed	4 (80.0%)	12 (80.0%)
Generic telephone messages	3 (60.0%)	8 (53.3%)
Billboards	1 (20.0%)	4 (26.7%)
Posters at pharmacy	5 (100%)	14 (93.3%)
Other	0	3 (20.0%)
None	0	1 (6.7%)

^a^ Strategies include those used for the marketing of any vaccine, not specific to the HPV vaccine.

**Table 4:** Pharmacists’ attitudes of Human Papillomavirus (HPV) and HPV vaccine, Number (%) ^a,b^.

Statement	Strongly Disagree	Somewhat Disagree	No Opinion/Unsure	Somewhat Agree	Strongly Agree
HPV vaccine is the best protection against cervical cancer. (*N* = 150)	2 (1.3)	5 (3.3)	15 (10.0)	57 (38.0)	**71 (47.3)**
HPV is a serious threat to a girl’s health.	10 (6.6)	9 (6.0)	13 (8.6)	50 (33.1)	**69 (45.7)**
HPV is a serious threat to a boy’s health.	12 (7.9)	15 (9.9)	40 (26.5)	**52 (34.4)**	32 (21.2)
I believe the optimal age to have a child vaccinated against HPV is age 11–12. (*N* = 150)	7 (4.7)	24 (16.0)	40 (26.7)	**49 (32.7)**	30 (20.0)
I believe the optimal age to have a child vaccinated against HPV is age 13–18. (*N* = 150)	13 (8.7)	19 (12.7)	41 (27.3)	**56 (37.3)**	21 (14.0)
Vaccinated children will not practice riskier sex behaviors.	29 (19.2)	24 (15.9)	32 (21.2)	**40 (26.5)**	26 (17.2)
HPV vaccine should be mandatory for all children age 11–12.	43 (28.5)	**49 (32.5)**	34 (22.5)	16 (10.6)	9 (6.0)
I have concerns about the safety of the HPV vaccine.	25 (16.6)	**43 (28.5)**	**43 (28.5)**	36 (24.8)	4 (2.6)
The side effects of HPV vaccine could outweigh the benefits.	25 (16.6)	**44 (29.1)**	33 (21.9)	36 (23.8)	13 (8.6)
I have concerns about the efficacy of the HPV vaccine. (*N* = 150)	32 (21.3)	**51 (34.0)**	35 (23.3)	23 (15.3)	9 (6.0)
I believe I would wait to encourage a child to be vaccinated against HPV until age 19–26.	**50 (33.1)**	49 (32.5)	40 (26.5)	9 (6.0)	3 (2.0)
I do not believe that children should be vaccinated against HPV until they are sexually active.	**64 (42.4)**	57 (37.7)	21 (13.9)	6 (4.0)	3 (2.0)
I do not believe in HPV vaccination because of religious or moral reasons.	**97 (64.2)**	26 (17.2)	25 (16.6)	3 (2.0)	0 (0)

^a^ The most frequently chosen responses/answers are bold for each question; ^b^ *N* = 151 unless otherwise stated.

**Table 5:** Pharmacists’ perceived system-related barriers to provision of HPV vaccine, Number (%) ^a^.

Statement on System Barriers ^b^	Not at All	A Little	Moderate	Very	Extremely
There are too few patients who want the HPV vaccine. (*N* = 147)	15 (10.2)	23 (15.6)	26 (17.7)	**46 (31.3)**	37 (25.2)
The failure of some insurance companies to cover the cost of vaccination. (*N* = 146)	20 (13.7)	14 (9.6)	32 (21.9)	**46 (31.5)**	34 (23.3)
The vaccine expiring before use. (*N* = 148)	24 (16.2)	18 (12.2)	26 (17.6)	**43 (29.1)**	37 (25.0)
The difficulty ensuring patients are completing the necessary 3 doses of the HPV vaccine. (*N* = 148)	25 (16.9)	26 (17.6)	38 (25.7)	**39 (26.4)**	20 (13.5)
The lack of adequate reimbursement for the HPV vaccination. (*N* = 146)	31 (21.2)	26 (17.8)	33 (22.6)	**34 (23.3)**	22 (15.1)
The cost of stocking the HPV vaccine. (*N* = 147)	**42 (28.6)**	27 (18.4)	29 (19.7)	34 (23.1)	15 (20.2)
The need to acquire a prescription from a physician to administer the HPV vaccine. (*N* = 147)	**54 (36.7)**	25 (17.0)	35 (23.8)	24 (16.3)	9 (6.1)
The amount of time it takes to talk to patients and/or parents about the HPV vaccine. (*N* = 147)	**69 (46.9)**	27 (18.4)	29 (19.7)	18 (12.2)	4 (2.7)
The refrigerator space needed to store the HPV vaccine. (*N* = 147)	**90 (61.2)**	27 (18.4)	20 (13.6)	9 (6.1)	1 (0.7)

^a^ The most frequently chosen responses/answers are bold for each question; ^b^ Respondents rated how likely each factor was to be a barrier in providing HPV vaccination services.

**Table 6:** Pharmacists’ perceived parent-related barriers to provision of HPV vaccine (*N* = 150), Number (%) ^a^.

Statement on Parent-Related Barriers ^b^	Strongly Disagree	Somewhat Disagree	Unsure	Somewhat Agree	Strongly Agree
Parents have concerns about the safety of the HPV vaccine.	0 (0)	6 (4.0)	26 (17.3)	**93 (62.0)**	25 (16.7)
Parents are concerned that by agreeing to have their children immunized, they are condoning premarital sex.	5 (3.3)	15 (10.0)	29 (19.3)	**83 (55.3)**	18 (12.0)
Parents have concerns about the efficacy of the HPV vaccine.	3 (2.0)	18 (12.0)	32 (21.3)	**83 (55.3)**	14 (9.3)
Parents lack adequate education / understanding about the HPV infection.	0 (0)	2 (1.3)	18 (12.0)	80 (53.3)	50 (33.3)
Parents believe their children are not at risk for HPV infection.	2 (1.3)	15 (10.0)	32 (21.3)	**80 (53.3)**	21 (14.0)
Parents are reluctant to discuss sexuality / sexually transmitted infections.	2 (1.3)	14 (9.3)	20 (13.3)	**76 (50.7)**	38 (25.3)
Parents believe their children are too young for the HPV vaccine.	2 (1.3)	8 (5.3)	42 (28.0)	**74 (49.3)**	24 (16.0)
Parents are concerned that their children will practice riskier sexual behaviors if they receive the HPV vaccine.	4 (2.7)	25 (16.7)	33 (22.0)	**73 (48.7)**	15 (10.0)
Parents believe the cost of the HPV vaccine is too high.	1 (0.7)	10 (6.7)	**59 (39.3)**	**59 (39.3)**	21 (14.0)
Parents will not consent to HPV vaccination.	4 (2.7)	23 (15.3)	**70 (46.7)**	51 (34.0)	2 (1.3)
Parents oppose HPV vaccination for moral or religious reasons.	5 (3.3)	27 (18.0)	**63 (42.0)**	48 (32.0)	7 (4.7)

^a^ The most frequently chosen responses/answers are bold for each question; ^b^ Respondents rated how likely each factor was to be a barrier in providing HPV vaccination services.
